# An association analysis of *HLA-DQB1* with narcolepsy without cataplexy and idiopathic hypersomnia with/without long sleep time in a Japanese population

**DOI:** 10.1038/hgv.2015.31

**Published:** 2015-09-17

**Authors:** Taku Miyagawa, Hiromi Toyoda, Takashi Kanbayashi, Aya Imanishi, Yohei Sagawa, Nozomu Kotorii, Tatayu Kotorii, Yuji Hashizume, Kimihiro Ogi, Hiroshi Hiejima, Yuichi Kamei, Akiko Hida, Masayuki Miyamoto, Azusa Ikegami, Yamato Wada, Masanori Takami, Yota Fujimura, Yoshiyuki Tamura, Naoto Omata, Yasuhiro Masuya, Hideaki Kondo, Shunpei Moriya, Hirokazu Furuya, Mitsuhiro Kato, Hiroto Kojima, Koichi Kashiwase, Hiroh Saji, Seik-Soon Khor, Maria Yamasaki, Jun Ishigooka, Yuji Wada, Shigeru Chiba, Naoto Yamada, Masako Okawa, Kenji Kuroda, Kazuhiko Kume, Koichi Hirata, Naohisa Uchimura, Tetsuo Shimizu, Yuichi Inoue, Yutaka Honda, Kazuo Mishima, Makoto Honda, Katsushi Tokunaga

**Affiliations:** 1 Department of Human Genetics, Graduate School of Medicine, The University of Tokyo, Tokyo, Japan; 2 Department of Neuropsychiatry, Akita University School of Medicine, Akita, Japan; 3 International Institute for Integrative Sleep Medicine (WPI-IIIS), University of Tsukuba, Ibaraki, Japan; 4 Department of Neuropsychiatry, Kurume University School of Medicine, Fukuoka, Japan; 5 Kotorii Isahaya Hospital, Nagasaki, Japan; 6 Sleep Disorder Center, National Center Hospital, National Center of Neurology and Psychiatry, Tokyo, Japan; 7 Department of Psychophysiology, National Institute of Mental Health, National Center of Neurology and Psychiatry, Tokyo, Japan; 8 Department of Neurology, Dokkyo Medical University, Tochigi, Japan; 9 Sleep Center, Kuwamizu Hospital, Kumamoto, Japan; 10 Department of Psychiatry, Hannan Hospital, Osaka, Japan; 11 Department of Psychiatry, Shiga University of Medical Science, Shiga, Japan; 12 Department of Psychiatry and Neurology, Asahikawa Medical University, Hokkaido, Japan; 13 Department of General Medicine, Faculty of Medicine, Shimane University, Shimane, Japan; 14 Department of Neuropsychiatry, Faculty of Medical Sciences, University of Fukui, Fukui, Japan; 15 Center for Sleep Medicine, Saiseikai Nagasaki Hospital, Nagasaki, Japan; 16 Department of Psychiatry, Tokyo Women's Medical University School of Medicine, Tokyo, Japan; 17 Department of Neurology, Neuro-Muscular Center, National Omuta Hospital, Fukuoka, Japan; 18 Department of Neurology, Kochi Medical School, Kochi University, Kochi, Japan; 19 Department of Pediatrics, Yamagata University Faculty of Medicine, Yamagata, Japan; 20 Department of Pediatrics, Showa University School of Medicine, Tokyo, Japan; 21 HLA Foundation Laboratory, Kyoto, Japan; 22 Department of HLA Laboratory, Japanese Red Cross Kanto-Koshinetsu Block Blood Center, Tokyo, Japan; 23 Department of Sleep Medicine, Shiga University of Medical Science, Shiga, Japan; 24 Japan Foundation for Neuroscience and Mental Health, Tokyo, Japan; 25 Department of Somnology, Tokyo Medical University, Tokyo, Japan; 26 Department of Stem Cell Biology, Institute of Molecular Genetics and Embryology, Kumamoto University, Kumamoto, Japan; 27 Department of Neuropharmacology, Graduate School of Pharmaceutical Sciences, Nagoya City University, Aichi, Japan; 28 Japan Somnology Center, Neuropsychiatric Research Institute, Tokyo, Japan; 29 Sleep Disorders Project, Department of Psychiatry and Behavioral Sciences, Tokyo Metropolitan Institute of Medical Science, Tokyo, Japan

## Abstract

Narcolepsy without cataplexy (NA w/o CA) (narcolepsy type 2) is a lifelong disorder characterized by excessive daytime sleepiness and rapid eye movement (REM) sleep abnormalities, but no cataplexy. In the present study, we examined the human leukocyte antigen *HLA-DQB1* in 160 Japanese patients with NA w/o CA and 1,418 control subjects. Frequencies of DQB1*06:02 were significantly higher in patients with NA w/o CA compared with controls (allele frequency: 16.6 vs. 7.8%, *P*=1.1×10^−7^, odds ratio (OR)=2.36; carrier frequency: 31.3 vs. 14.7%, *P*=7.6×10^−8^, OR=2.64). Distributions of *HLA-DQB1* alleles other than DQB1*06:02 were compared between NA w/o CA and narcolepsy with cataplexy (NA-CA) to assess whether the genetic backgrounds of the two diseases have similarities. The distribution of the *HLA-DQB1* alleles in DQB1*06:02-negative NA w/o CA was significantly different from that in NA-CA (*P*=5.8×10^−7^). On the other hand, the patterns of the *HLA-DQB1* alleles were similar between DQB1*06:02-positive NA w/o CA and NA-CA. *HLA-DQB1* analysis was also performed in 186 Japanese patients with idiopathic hypersomnia (IHS) with/without long sleep time, but no significant associations were observed.

The 2nd Edition of the International Classification of Sleep Disorders (ICSD-2), in the category of hypersomnia of central origin, subdivides narcolepsy into two groups: narcolepsy with cataplexy (NA-CA) and narcolepsy without cataplexy (NA w/o CA). NA w/o CA is characterized by excessive daytime sleepiness and abnormal manifestations of rapid eye movement (REM) sleep in common with NA-CA, but no cataplexy. Patients with NA w/o CA have frequent sleep-onset REM periods, as do those with NA-CA, as revealed by performance of the multiple sleep latency test. A population-based study suggested that the prevalence of NA w/o CA is 36% of the prevalence of narcolepsy as a whole, corresponding to a point prevalence of 0.02%.^1^ NA-CA is tightly associated with HLA-DQB1*06:02 and orexin (hypocretin) deficiency. Almost all patients with NA-CA in many populations consistently carry DQB1*06:02, while approximately 12% of Japanese, 25% of Caucasian and 38% of African American healthy individuals are DQB1*06:02-positive.^2–4^ Low levels of orexin A in cerebrospinal fluid (CSF) (<110 pg/ml) are commonly observed in patients with NA-CA.^5,6^ Regarding NA w/o CA, positivity of HLA-DQB1*06:02 (30–50%) is also higher than that in the general population,^7–10^ but less than that in NA-CA. However, only approximately 20% of patients with NA w/o CA have low levels of CSF orexin A,^6,10^ indicating that the etiology of the majority of NA w/o CA is still unknown.

There have been a number of studies of *HLA* in NA-CA; results indicated that *HLA-DQB1* alleles other than DQB1*06:02 modulate susceptibility or resistance to NA-CA. DQB1*06:01 and DQB1*05:01 in the Korean and Japanese populations and DQB1*06:03 in European populations are protective against NA-CA,^2,7,11–14^ whereas individuals with DQB1*03:01 and DQB1*03:02 are at an increased risk.^2,7,11,13–17^ In the present study, to test for associations of *HLA-DQB1* alleles in NA w/o CA, we performed an association study for *HLA-DQB1* in 160 Japanese patients with NA w/o CA and 1,418 control subjects.

Idiopathic hypersomnia (IHS) is a sleep disorder of presumed central nervous system origin that is associated with excessive daytime sleepiness consisting of prolonged non-REM sleep episodes. Daytime naps of IHS patients tend to be longer and less refreshing than those of NA-CA patients. IHS is a rare disease, representing 8:10 to 1:10 patients with NA-CA. This suggests that the prevalence of IHS approximates 0.005%.^18^ The ICSD-2 describes two clinical forms of IHS by the difference in nocturnal sleep time: IHS with long sleep time (IHS-LST) and IHS without long sleep time (IHS w/o LST). The nocturnal sleep time of IHS-LST is prolonged to at least 10 h, while that of IHS w/o LST is either normal or slightly prolonged (less than 10 h). CSF orexin A levels in IHS are normal.^6^ The cause and pathogenesis of IHS remain largely unknown. NA w/o CA and IHS w/o LST have several common characteristics except for REM-related symptoms. Distinguishing NA w/o CA and IHS w/o LST is impossible without the multiple sleep latency test to identify sleep-onset REM periods. According to the ICSD-2, the diagnosis is based on the number of sleep-onset REM periods, two or more in the former and less than two in the latter. In the present study, we tested whether *HLA-DQB1* alleles have an influence on susceptibility to IHS w/o LST and IHS-LST.

A total of 346 Japanese patients and 1,418 Japanese healthy controls were included in this study. NA w/o CA, IHS w/o LST and IHS-LST were diagnosed according to the ICSD-2 criteria. The patient groups consisted of NA w/o CA (*n*=160), IHS w/o LST (*n*=118) and IHS-LST (*n*=68). We utilized *HLA* data of healthy individuals, who have been previously studied for disease association analyses.^17,19,20^ In addition, to assess genetic similarities between the above hypersomnia disorders and NA-CA, *HLA-DQB1* data from 664 patients with NA-CA were utilized.^17^ All of the patients and controls were mainland Japanese and gave written informed consent. This study was approved by the local institutional review boards at participating institutions. Typing for the *HLA-DQB1* locus was performed by a Luminex Multi-Analyte Profiling system (xMAP) with WAKFlow HLA typing kits (Wakunaga Pharmaceutical, Wakunaga, Hiroshima, Japan). Comparisons of frequencies were performed using the Chi-square test or Fisher’s Exact test as appropriate. To account for multiple testing, the significance level was adjusted by the number of *HLA* alleles with allele frequencies no less than 0.5% in controls (12 for *HLA-DQB1* alleles). The significance level was set to be *P*<4.2×10^−3^ (0.05/12). If any of the four cells was zero, the Woolf-Haldane correction was applied (adding 0.5 to all cells). An association analysis controlling for the effects of DQB1*06:02 was needed to test the other *HLA-DQB1* alleles. Specifically, the analysis was performed using counts of the other *HLA-DQB1* alleles remaining after excluding allele counts of DQB1*06:02 from both cases and controls, which is the relative predispositional effects method.^21^ Briefly, frequencies and ORs were calculated for the alleles carried by the non-DQB1*06:02 chromosomes. To determine whether there was a different allelic distribution between two groups, the overall frequency distribution of *HLA-DQB1* alleles in one group was compared with the distribution in another group by using the global Chi-square test with 12 degrees-of-freedom.

*HLA-DQB1* allele frequencies of 160 patients with NA w/o CA and 1,418 control subjects were determined, and are shown in Table 1. DQB1*06:02 was significantly associated with NA w/o CA (allele frequency: 16.6 vs. 7.8% in controls, *P*=1.1×10^−7^, OR=2.36). Regarding carrier frequencies for *HLA-DQB1*, 31.3% of the patients carried DQB1*06:02, vs. 14.7% of controls (*P*=7.6×10^−8^, OR=2.64) (Supplementary Table 1). We tested whether there were other independent *HLA-DQB1* associations aside from DQB1*06:02 using the relative predispositional effect method.^21^ DQB1*06:04 showed a marginal association (*P*=5.9×10^−3^, OR=1.80) (Table 2). However, the *P* value did not reach the threshold corrected for multiple comparisons. NA w/o CA was subdivided into DQB1*06:02-positive and -negative groups for further analyses, because previous studies suggested that these two disease groups have different etiology, clinical characteristics and electroencephalographic findings.^10,22–24^ The analyses were performed by controlling for the effects of DQB1*06:02 (Table 2). No significant association with *HLA-DQB1* alleles was found in DQB1*06:02-positive NA w/o CA. DQB1*06:04 was nominally associated with DQB1*06:02-negative NA w/o CA patients (*P*=5.6×10^−3^, OR=1.88).

Next, we tested whether *HLA-DQB1* allele distributions (except for DQB1*06:02) in DQB1*06:02-positive and -negative NA w/o CA patients showed similarities with those of NA-CA patients (Table 2 and Supplementary Figure 1). The allele distribution of DQB1*06:02-negative NA w/o CA was significantly different from that of NA-CA (*P*=5.8×10^−7^). On the other hand, no significant difference was observed in the distribution between DQB1*06:02-positive NA w/o CA and NA-CA (*P*=0.97). When we focused on *HLA-DQB1* alleles with frequencies no less than 5% in DQB1*06:02-positive NA w/o CA, all the ORs of the alleles were found in the same direction as those of NA-CA (Table 2). These results suggest that a common etiological pathway might exist for DQB1*06:02-positive NA w/o CA and NA-CA, whereas DQB1*06:02-negative NA w/o CA has a different etiological pathway from NA-CA. Orexin A in the CSF of typical NA-CA patients is known to be reduced or undetectable.^5,6^ A minority of NA w/o CA patients have low concentrations of CSF orexin A levels, although a majority of NA w/o CA patients have normal concentrations;^6,10^ thus, an etiologic heterogeneity in NA w/o CA is suggested. In addition, most of the NA w/o CA patients with low CSF orexin A are DQB1*06:02-positive, as in NA-CA.^6,10^ In contrast, DQB1*06:02 positivity of patients with normal CSF orexin A is not higher than that seen in the general population.^10^ Therefore, the heterogeneity in NA w/o CA might be divided by DQB1*06:02 positivity, and DQB1*06:02-positive NA w/o CA might have the same etiology as NA-CA from the view point of orexin deficiency. In addition, clinical characteristics and electroencephalographic findings have been reported to be different between DQB1*06:02-positive and -negative NA w/o CA groups.^23,24^ These findings correspond well to our result.

Patients with IHS w/o LST and IHS-LST were also typed for *HLA-DQB1* in the present study. *HLA-DQB1* allele and carrier frequencies are shown in Table 3 and Supplementary Table 2, respectively. DQB1*06:02, known to be associated with NA-CA and NA w/o CA, was not associated with IHS w/o LST or IHS-LST. Although DQB1*05:02 (*P*=6.3×10^−3^) and DQB1*03:01 (*P*=0.04) showed nominally significant associations with IHS-LST, there were no *HLA-DQB1* alleles that reached the threshold after correction for multiple comparisons. The similarity of *HLA-DQB1* allele distribution between NA-CA and IHS w/o LST or IHS-LST was tested after controlling for the effects of DQB1*06:02, and significant differences were found: for IHS w/o LST: *P*=2.1×10^−4^ and for IHS-LST: *P*=2.2×10^−8^. Taken together, these results indicate that IHS w/o LST and IHS-LST are caused by different etiological genetic factors than those that give rise to NA-CA.

The International Classification of Sleep Disorders was recently revised for the 3rd Edition (ICSD-3). When we had recruited patient samples, the 2nd edition (ICSD-2) was used. Main differences between the ICSD-2 and ICSD-3 regarding NA w/o CA, IHS w/o LST and IHS-LST are as follows. The terminology has been changed from ‘narcolepsy without cataplexy (NA w/o CA)’ to ‘narcolepsy type 2’. The concept of NA w/o CA and narcolepsy type 2 is almost the same. IHS w/o LST and IHS-LST were unified to ‘idiopathic hypersomnia (IHS)’ regardless of the extension of the sleep time. Therefore, we combined our IHS w/o LST and IHS-LST data to analyze the *HLA-DQB1* data as IHS (Supplementary Tables 3 and 4). As a result, no significant association was observed between each *HLA-DQB1* allele and IHS. Distribution of *HLA-DQB1* alleles in IHS was also significantly different from that of NA-CA, even after the effect of *DQB1-06:02* was controlled (*P*=8.6×10^−9^).

To conclude, the association of DQB1*06:02 in NA w/o CA was confirmed. Our results also suggested an immunological pathogenesis of DQB1*06:02-positive NA w/o CA, which is similar to that of NA-CA. DQB1*06:02-negative NA w/o CA, IHS w/o LST and IHS-LST may have a different etiology, which is not well understood.

## Figures and Tables

**Table 1:** *HLA-DQB1* allele frequencies of patients with NA w/o CA

*DQB1*	*NA w/o CA*	*(2n=320)*	*Control*	*(2n=2836)*	*OR*	P
	*No.*	*%*	*No.*	*%*		
*02:01*	1	0.3	11	0.4	0.81	1.00
*03:01*	29	9.1	334	11.8	0.75	0.15
*03:02*	27	8.4	264	9.3	0.90	0.61
*03:03*	46	14.4	450	15.9	0.89	0.49
*04:01*	42	13.1	374	13.2	0.99	0.97
*04:02*	12	3.8	118	4.2	0.90	0.73
*05:01*	22	6.9	191	6.7	1.02	0.92
*05:02*	3	0.9	63	2.2	0.42	0.13
*05:03*	10	3.1	106	3.7	0.83	0.58
*06:01*	45	14.1	515	18.2	0.74	0.07
*06:02*	53	16.6	220	7.8	2.36	1.1.E−07
*06:03*	1	0.3	16	0.6	0.55	1.00
*06:04*	28	8.8	160	5.6	1.60	0.03
*06:09*	1	0.3	14	0.5	0.91	1.00

Abbreviations: NA w/o CA, narcolepsy without cataplexy; OR, odds ratio.

**Table 2:** Frequencies of *HLA-DQB1* alleles after removal of DQB1*06:02 effects

*DQB1 alleles*	*NA w/o CA*				DQB1*06:02 (+) NA w/o CA				DQB1*06:02 (−) NA w/o CA				*Control*		*NA-CA*			
	*No.*	*%*	*OR*	P^a^	*No.*	*%*	*OR*	P^a^	*No.*	*%*	*OR*	P^a^	*No.*	*%*	*No.*	*%*	*OR*	P^a^
*02:01*	1	0.4	0.89	1.00	0	0.0	2.38	1.00	1	0.5	1.08	1.00	11	0.4	5	0.9	1.78	0.22
*03:01*	29	10.9	0.83	0.37	9	19.1	1.62	0.20	20	9.1	0.68	0.11	334	12.8	98	16.9	1.38	5.7.E−03
*03:02*	27	10.1	1.00	0.99	6	12.8	1.30	0.47	21	9.5	0.94	0.80	264	10.1	107	18.4	1.97	2.5.E−09
*03:03*	46	17.2	1.00	0.99	8	17.0	0.99	0.97	38	17.3	1.00	0.98	450	17.2	74	12.8	0.73	0.02
*04:01*	42	15.7	1.12	0.53	9	19.1	1.42	0.35	33	15.0	1.06	0.78	374	14.3	112	19.3	1.43	1.2.E−03
*04:02*	12	4.5	1.00	0.99	1	2.1	0.46	0.72	11	5.0	1.11	0.74	118	4.5	20	3.4	0.77	0.27
*05:01*	22	8.2	1.14	0.58	2	4.3	0.56	0.58	20	9.1	1.27	0.33	191	7.3	20	3.4	0.48	1.5.E−03
*05:02*	3	1.1	0.46	0.18	1	2.1	0.88	1.00	2	0.9	0.37	0.15	63	2.4	28	4.8	1.80	4.4.E−03
*05:03*	10	3.7	0.92	0.81	1	2.1	0.51	1.00	9	4.1	1.01	0.98	106	4.1	21	3.6	0.79	0.31
*06:01*	45	16.9	0.83	0.27	6	12.8	0.60	0.24	39	17.7	0.88	0.48	515	19.7	49	8.4	0.39	1.4.E−10
*06:03*	1	0.4	0.61	1.00	0	0.0	1.66	1.00	1	0.5	0.74	1.00	16	0.6	1	0.2	0.25	0.26
*06:04*	28	10.5	1.80	5.9.E−03	4	8.5	1.43	0.50	24	10.9	1.88	5.6.E−03	160	6.1	42	7.2	1.10	0.58
*06:09*	1	0.4	0.70	1.00	0	0.0	1.89	1.00	1	0.5	0.85	1.00	14	0.5	3	0.5	1.50	0.46
Global *P* value versus NA-CA^b^	4.3.E−06				0.97				5.8E−07				1.2.E−17					

Abbreviations: NA-CA, narcolepsy with cataplexy; NA w/o CA, narcolepsy without cataplexy; OR, odds ratio.

NA w/o CA: *n*=267, DQB1*06:02 (+) NA w/o CA: *n*=47, DQB1*06:02 (−) NA w/o CA: *n*=220, Control: *n*=2616, NA-CA: *n*=580.

a *P* values were calculated for comparisons between disease and control groups.

b The overall frequency distribution of *HLA-DQB1* alleles in NA-CA group was compared with that in another group using the global Chi-square test.

**Table 3:** *HLA-DQB1* allele frequencies of patients with IHS w/o LST and IHS-LST

*DQB1*	*IHS w/o LST*	*(2n=236)*			*IHS-LST*	*(2n=136)*			*Control*	*(2n=2836)*
	*No.*	*%*	*OR*	P	*No.*	*%*	*OR*	P	*No.*	*%*
*02:01*	0	0.0	0.52	1.00	0	0.0	0.90	1.00	11	0.4
*03:01*	33	14.0	1.22	0.32	8	5.9	0.47	0.04	334	11.8
*03:02*	26	11.0	1.21	0.39	10	7.4	0.77	0.44	264	9.3
*03:03*	34	14.4	0.89	0.55	19	14.0	0.86	0.55	450	15.9
*04:01*	35	14.8	1.15	0.48	13	9.6	0.70	0.22	374	13.2
*04:02*	9	3.8	0.91	0.80	4	2.9	0.70	0.48	118	4.2
*05:01*	19	8.1	1.21	0.44	11	8.1	1.22	0.54	191	6.7
*05:02*	5	2.1	0.95	0.92	8	5.9	2.75	6.3.E−03	63	2.2
*05:03*	8	3.4	0.90	0.79	8	5.9	1.61	0.20	106	3.7
*06:01*	39	16.5	0.89	0.53	33	24.3	1.44	0.07	515	18.2
*06:02*	16	6.8	0.86	0.59	12	8.8	1.15	0.65	220	7.8
*06:03*	2	0.8	1.51	0.64	1	0.7	1.31	0.55	16	0.6
*06:04*	9	3.8	0.66	0.24	8	5.9	1.05	0.91	160	5.6
*06:09*	1	0.4	0.86	1.00	1	0.7	1.49	0.51	14	0.5

Abbreviations: IHS-LST, idiopathic hypersomnia with long sleep time; IHS w/o LST, idiopathic hypersomnia without long sleep time; OR, odds ratio.
