# Supplementary material for: An association analysis of HLA-DQB1 with narcolepsy without cataplexy and idiopathic hypersomnia with/without long sleep time in a Japanese population
Source: Hum Genome Var. 2015 Sep 17;2:15031–. doi: 10.1038/hgv.2015.31 (PMC4785567; doi:10.1038/hgv.2015.31)
Supplement: Supplementary Tables [file hgv201531-s1.doc]

Supplementary Table 1. *HLA-DQB1* carrier frequencies of patients with NA w/o CA

|  | NA w/o CA | (n=160) |  | Control | (n=1418) |  |  |
| --- | --- | --- | --- | --- | --- | --- | --- |
| *DQB1* | No. | % |  | No. | % | OR | P |
| *02:01* | 1 | 0.6% |  | 11 | 0.8% | 0.80 | 1.00 |
| *03:01* | 28 | 17.5% |  | 318 | 22.4% | 0.73 | 0.15 |
| *03:02* | 26 | 16.3% |  | 252 | 17.8% | 0.90 | 0.63 |
| *03:03* | 42 | 26.3% |  | 413 | 29.1% | 0.87 | 0.45 |
| *04:01* | 39 | 24.4% |  | 340 | 24.0% | 1.02 | 0.91 |
| *04:02* | 11 | 6.9% |  | 117 | 8.3% | 0.82 | 0.55 |
| *05:01* | 20 | 12.5% |  | 184 | 13.0% | 0.96 | 0.86 |
| *05:02* | 3 | 1.9% |  | 62 | 4.4% | 0.42 | 0.13 |
| *05:03* | 10 | 6.3% |  | 104 | 7.3% | 0.84 | 0.62 |
| *06:01* | 43 | 26.9% |  | 458 | 32.3% | 0.77 | 0.16 |
| *06:02* | 50 | 31.3% |  | 208 | 14.7% | 2.64 | 7.6.E-08 |
| *06:03* | 1 | 0.6% |  | 16 | 1.1% | 0.55 | 1.00 |
| *06:04* | 26 | 16.3% |  | 151 | 10.6% | 1.63 | 0.03 |
| *06:09* | 1 | 0.6% |  | 13 | 0.9% | 0.98 | 1.00 |

OR, odds ratio

Supplementary Table 2. *HLA-DQB1* carrier frequencies of patients with IHS w/o LST and IHS-LST

|  | IHS w/o LST | (n=118) |  |  |  | IHS-LST | (n=68) |  |  |  | Control | (n=1418) |
| --- | --- | --- | --- | --- | --- | --- | --- | --- | --- | --- | --- | --- |
| *DQB1* | No. | % | OR | P |  | No. | % | OR | P |  | No. | % |
| *02:01* | 0 | 0.0% | 0.52 | 1.00 |  | 0 | 0.0% | 0.89 | 1.00 |  | 11 | 0.8% |
| *03:01* | 31 | 26.3% | 1.23 | 0.34 |  | 8 | 11.8% | 0.46 | 0.04 |  | 318 | 22.4% |
| *03:02* | 23 | 19.5% | 1.12 | 0.64 |  | 9 | 13.2% | 0.71 | 0.34 |  | 252 | 17.8% |
| *03:03* | 31 | 26.3% | 0.87 | 0.51 |  | 15 | 22.1% | 0.69 | 0.21 |  | 413 | 29.1% |
| *04:01* | 32 | 27.1% | 1.18 | 0.44 |  | 13 | 19.1% | 0.75 | 0.36 |  | 340 | 24.0% |
| *04:02* | 9 | 7.6% | 0.92 | 0.81 |  | 4 | 5.9% | 0.69 | 0.49 |  | 117 | 8.3% |
| *05:01* | 19 | 16.1% | 1.29 | 0.34 |  | 11 | 16.2% | 1.29 | 0.45 |  | 184 | 13.0% |
| *05:02* | 5 | 4.2% | 0.97 | 0.94 |  | 8 | 11.8% | 2.92 | 4.9.E-03 |  | 62 | 4.4% |
| *05:03* | 6 | 5.1% | 0.68 | 0.36 |  | 8 | 11.8% | 1.68 | 0.18 |  | 104 | 7.3% |
| *06:01* | 34 | 28.8% | 0.85 | 0.44 |  | 27 | 39.7% | 1.38 | 0.20 |  | 458 | 32.3% |
| *06:02* | 16 | 13.6% | 0.91 | 0.74 |  | 10 | 14.7% | 1.00 | 0.99 |  | 208 | 14.7% |
| *06:03* | 2 | 1.7% | 1.51 | 0.64 |  | 1 | 1.5% | 1.31 | 0.55 |  | 16 | 1.1% |
| *06:04* | 9 | 7.6% | 0.69 | 0.30 |  | 8 | 11.8% | 1.12 | 0.77 |  | 151 | 10.6% |
| *06:09* | 1 | 0.8% | 0.92 | 1.00 |  | 1 | 1.5% | 1.61 | 0.48 |  | 13 | 0.9% |

OR, odds ratio

Supplementary Table 3. *HLA-DQB1* allele frequencies of patients with IHS

|  | IHS | (2n=372) |  | Control | (2n=2836) |  |  |
| --- | --- | --- | --- | --- | --- | --- | --- |
| *DQB1* | No. | % |  | No. | % | OR | P |
| *02:01* | 0 | 0.0% |  | 11 | 0.4% | 0.33 | 0.63 |
| *03:01* | 41 | 11.0% |  | 334 | 11.8% | 0.93 | 0.67 |
| *03:02* | 36 | 9.7% |  | 264 | 9.3% | 1.04 | 0.82 |
| *03:03* | 53 | 14.2% |  | 450 | 15.9% | 0.88 | 0.42 |
| *04:01* | 48 | 12.9% |  | 374 | 13.2% | 0.98 | 0.88 |
| *04:02* | 13 | 3.5% |  | 118 | 4.2% | 0.83 | 0.54 |
| *05:01* | 30 | 8.1% |  | 191 | 6.7% | 1.21 | 0.34 |
| *05:02* | 13 | 3.5% |  | 63 | 2.2% | 1.59 | 0.13 |
| *05:03* | 16 | 4.3% |  | 106 | 3.7% | 1.16 | 0.59 |
| *06:01* | 72 | 19.4% |  | 515 | 18.2% | 1.08 | 0.58 |
| *06:02* | 28 | 7.5% |  | 220 | 7.8% | 0.97 | 0.88 |
| *06:03* | 3 | 0.8% |  | 16 | 0.6% | 1.43 | 0.48 |
| *06:04* | 17 | 4.6% |  | 160 | 5.6% | 0.80 | 0.39 |
| *06:09* | 2 | 0.5% |  | 14 | 0.5% | 1.09 | 0.71 |

OR, odds ratio

Supplementary Table 4. *HLA-DQB1* carrier frequencies of patients with IHS

|  | IHS | (n=186) |  | Control | (n=1418) |  |  |
| --- | --- | --- | --- | --- | --- | --- | --- |
| *DQB1* | No. | % |  | No. | % | OR | P |
| *02:01* | 0 | 0.0% |  | 11 | 0.8% | 0.33 | 0.63 |
| *03:01* | 39 | 21.0% |  | 318 | 22.4% | 0.92 | 0.65 |
| *03:02* | 32 | 17.2% |  | 252 | 17.8% | 0.96 | 0.85 |
| *03:03* | 46 | 24.7% |  | 413 | 29.1% | 0.80 | 0.21 |
| *04:01* | 45 | 24.2% |  | 340 | 24.0% | 1.01 | 0.95 |
| *04:02* | 13 | 7.0% |  | 117 | 8.3% | 0.84 | 0.55 |
| *05:01* | 30 | 16.1% |  | 184 | 13.0% | 1.29 | 0.23 |
| *05:02* | 13 | 7.0% |  | 62 | 4.4% | 1.64 | 0.11 |
| *05:03* | 14 | 7.5% |  | 104 | 7.3% | 1.03 | 0.92 |
| *06:01* | 61 | 32.8% |  | 458 | 32.3% | 1.02 | 0.89 |
| *06:02* | 26 | 14.0% |  | 208 | 14.7% | 0.95 | 0.80 |
| *06:03* | 3 | 1.6% |  | 16 | 1.1% | 1.44 | 0.48 |
| *06:04* | 17 | 9.1% |  | 151 | 10.6% | 0.84 | 0.53 |
| *06:09* | 2 | 1.1% |  | 13 | 0.9% | 1.17 | 0.69 |

OR, odds ratio
