# Supplementary material for: An association analysis of HLA-DQB1 with narcolepsy without cataplexy and idiopathic hypersomnia with/without long sleep time in a Japanese population
Source: Hum Genome Var. 2015 Sep 17;2:15031–. doi: 10.1038/hgv.2015.31 (PMC4785567; doi:10.1038/hgv.2015.31)
Supplement: Supplementary Figure 1 [file hgv201531-s2.ppt]

## Slide 1
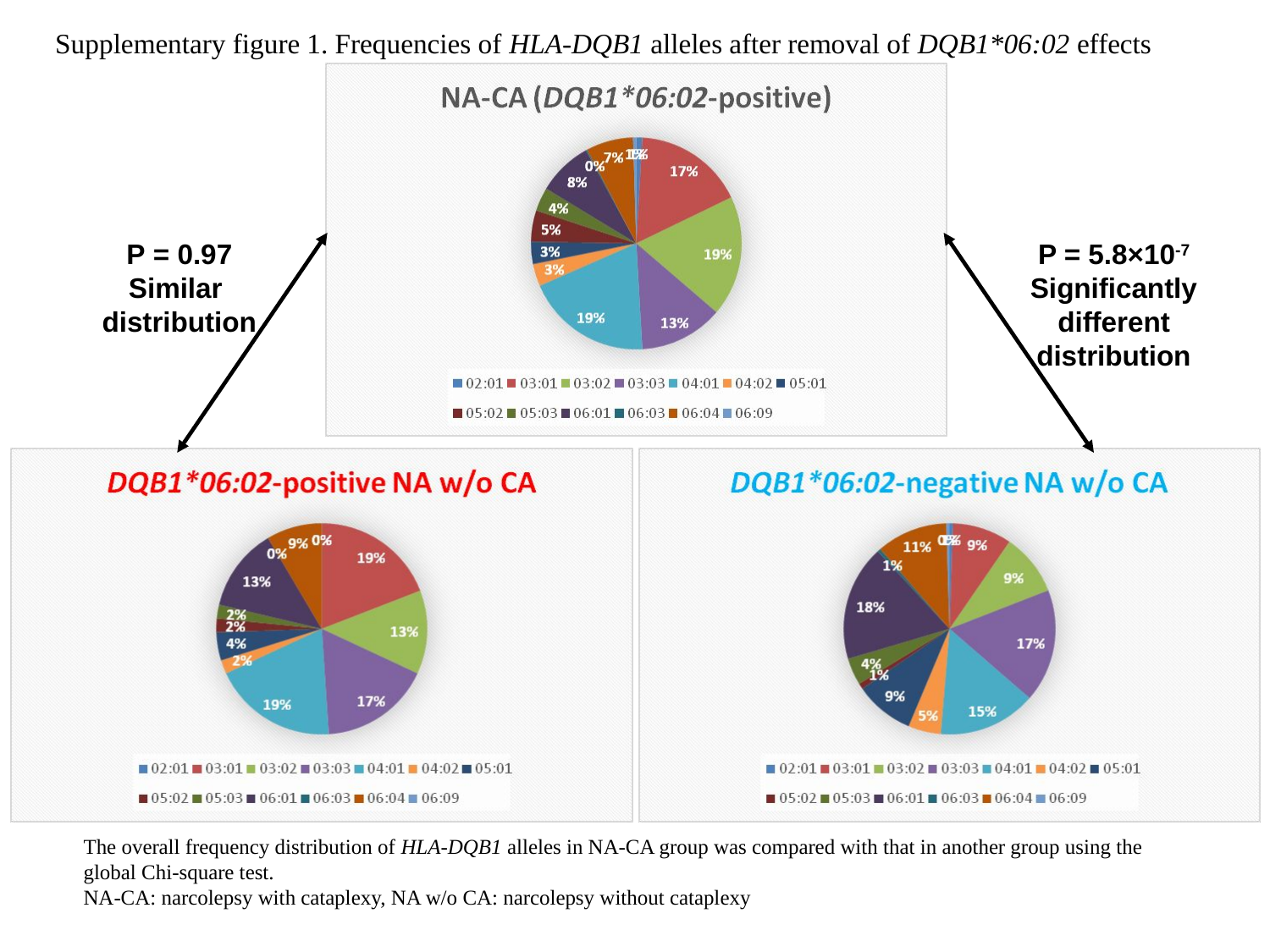

Supplementary figure 1. Frequencies of HLA-DQB1 alleles after removal of DQB1*06:02 effects
P = 0.97
Similar
distribution
P = 5.8×10^-7^
Significantly different distribution
The overall frequency distribution of HLA-DQB1 alleles in NA-CA group was compared with that in another group using the global Chi-square test.
NA-CA: narcolepsy with cataplexy, NA w/o CA: narcolepsy without cataplexy
